# Reply to: Whole‐Body Magnetic Resonance Imaging in Camurati–Engelmann Disease

**DOI:** 10.1002/jbm4.10355

**Published:** 2020-03-25

**Authors:** Tim Cundy, Peter Hughes

**Affiliations:** ^1^ Department of Endocrinology Greenlane Clinical Centre Auckland; ^2^ Department of Radiology Auckland City Hospital Auckland

**To The Editor**

We thank Dr Corrêa and colleagues for their interest in our paper. The whole‐body MR images they show from a 7‐year‐old child with active Camaurati–Englemann disease are certainly very striking. It would be interesting to see imaging of late‐stage disease when the activity—as judged by scintigraphy—has decreased. T2‐short tau inversion recovery imaging from whole‐body MRI could be very useful in evaluating the effectiveness of potential medical therapies, such as angiotensin receptor blockers or glucocorticoids.

